# Group-based developmental BMI trajectories, polycystic ovary syndrome, and gestational diabetes: a community-based longitudinal study

**DOI:** 10.1186/s12916-017-0957-7

**Published:** 2017-11-06

**Authors:** Nadira Sultana Kakoly, Arul Earnest, Lisa J. Moran, Helena J. Teede, Anju E. Joham

**Affiliations:** 1 Monash Centre for Health Research and Implementation, School of Public Health and Preventive Medicine, Monash University, Clayton, Victoria 3168 Australia; 2 Diabetes and Vascular Medicine Unit, Monash Health, Clayton, Victoria 3168 Australia; 3 Monash Partners Academic Health Sciences Centre, Melbourne, Victoria Australia; 4 School of Public Health and Preventive Medicine, Monash University, Locked bag 29, Monash Medical Centre, Clayton, Victoria 3168 Australia

**Keywords:** Polycystic ovary syndrome, BMI, Longitudinal, Gestational diabetes, Latent-curve analysis

## Abstract

**Background:**

Obesity is common in young women, increasing insulin resistance (IR) and worsening pregnancy complications, including gestational diabetes (GDM). Women with polycystic ovary syndrome (PCOS) are commonly obese, which aggravates the severity of PCOS clinical expression. Relationships between these common insulin-resistant conditions, however, remain unclear.

**Methods:**

We conducted a secondary analysis of the Australian Longitudinal Study on Women’s Health (ALSWH) database, including data from 8009 women aged 18–36 years across six surveys. We used latent-curve growth modelling to identify distinct body mass index (BMI) trajectories and multinomial logistic regression to explore sociodemographic and health variables characterizing BMI group membership. Logistic regression was used to assess independent risk of GDM.

**Results:**

A total of 662 women (8.29%, 95% CI 7.68–8.89) reported PCOS. Three distinct BMI trajectories emerged, namely low stable (LSG) (63.8%), defined as an average trajectory remaining at ~25 kg/m^2^; moderately rising (MRG) (28.8%), a curvilinear trajectory commencing in a healthy BMI and terminating in the overweight range; and high-rising (HRG) (7.4%), a curvilinear trajectory starting and terminating in the obese range. A high BMI in early reproductive life predicted membership in higher trajectories. The HRG BMI trajectory was independently associated with GDM (OR 2.50, 95% CI 1.80–3.48) and was a stronger correlate than PCOS (OR 1.89, 95% CI 1.41–2.54), maternal age, socioeconomic status, or parity.

**Conclusion:**

Our results suggest heterogeneity in BMI change among Australian women of reproductive age, with and without PCOS. Reducing early adult life weight represents an ideal opportunity to intervene at an early stage of reproductive life and decreases the risk of long-term metabolic complications such as GDM.

## Background

Worldwide, there is an alarming rise in weight gain and obesity [1], fueled by adverse lifestyle risk factors [2]. This increasing trend is especially concerning among women of reproductive age in developed countries like Australia, where up to 44% of women entering pregnancy are overweight or obese [3]. Obesity increases insulin resistance (IR), worsens pregnancy complications such as gestational diabetes (GDM) [4], and exacerbates other common insulin-resistant conditions including polycystic ovary syndrome (PCOS) [5]. Obesity, PCOS, and GDM all impose additional risks in pregnancy and present long-term health risks for mothers and their progeny. However, little is known about the relationships between these common insulin-resistant conditions, with prior meta-analyses exploring relationships between obesity and PCOS or GDM and PCOS showing significant heterogeneity [6].

PCOS is a common endocrine disorder underpinned by hyperandrogenism and IR that affects up to 18% of women of reproductive age [7]. The syndrome is associated with a range of metabolic and pregnancy complications, including obesity, IR, type 2 diabetes (T2DM), GDM, and preeclampsia [7]. PCOS is further aggravated by extrinsic or obesity-related IR, increasing the prevalence and severity of the condition [8]. GDM is defined as carbohydrate intolerance first recognized during pregnancy and is a common pregnancy-related complication, affecting approximately 7% of women [9, 10]. GDM with gestational weight gain has significant neonatal and maternal complications, including large for gestational age, macrosomia [11], premature deliveries, and stillbirth, as well as long-term risks such as the development of T2DM [12]. While existing studies recognize GDM as a complication of PCOS, with an approximately three-fold risk, most have not adjusted for important confounders, including obesity or body mass index (BMI) [13, 14].

Studies in the general population that have longitudinally explored BMI or weight change have mostly used conventional growth modeling, assuming the population is homogenous with one weight trajectory [15]. Studies using group-based trajectory modeling (GBTM) to examine changes in BMI in association with lifestyle-related disorders, such as T2DM and cardiovascular disease (CVD), typically showed heterogeneity of the disorders in terms of differing pathophysiological disease pathways among different segments of the study population categorized according to change in BMI over time. Dhana et al. [16] identified three distinct trajectories of BMI among participants who developed CVD. The majority of participants who developed the disease were characterized with a stable BMI over time, suggesting BMI alone to be a poor indicator for identifying populations at risk of CVD. Conversely, Vistisen et al. [17] explored patterns of BMI change over time among 6705 white British participants before the development of T2DM and found three distinct BMI trajectories associated with differing rates of change in IR and cardiometabolic risk factors, with the majority of patients showing modest weight gain prior to diagnosis.

There is evidence to suggest that a higher BMI is more commonly seen in women with PCOS, with BMI over time increasing more in women with PCOS compared to women without [18, 19]. Currently, there is no literature examining empirically derived categorizations of BMI taking into account change over time both among women of reproductive age in general or specifically among higher-risk women, including those with PCOS. GBTM captures BMI change over time by identifying and empirically grouping individuals into latent classes, thus providing insight into BMI trajectory determinants and taking into account population heterogeneity, allowing group comparisons [20].

To our knowledge, no studies have examined latent BMI trajectory groups in community-based cohorts of women of reproductive age or among women with PCOS. Furthermore, there is no prior research exploring the relationship between BMI trajectories, their sociodemographic predictors, PCOS, and GDM development. Therefore, we aimed to address these knowledge gaps in a large national longitudinal cohort study of community-based women of reproductive age.

## Methods

### Study type

We conducted secondary analysis of the Australian Longitudinal Study on Women’s Health (ALSWH) database, spanning 16 years of data collected from six surveys. The ALSWH is a longitudinal population-based survey examining the health of more than 58,000 Australian women. In 1996, the ALSWH first collected mailed survey data from three age cohorts of Australian women. The study randomly selected women from the national health insurance scheme (Medicare) database, which includes records of all permanent residents of Australia. Women were intentionally oversampled from rural and remote areas. Data was collected based on mailed surveys. Prior to this study, this dataset was analyzed primarily in cross-sectional studies on PCOS and health outcomes; further details of the methods used and sample characteristics have been documented previously [21]. Additional details are also available on the ALSWH website (http://www.alswh.org.au). The Human Research Ethics Committees of the University of Newcastle and the University of Queensland approved the study methods.

### Participants

**Fig. 1:**
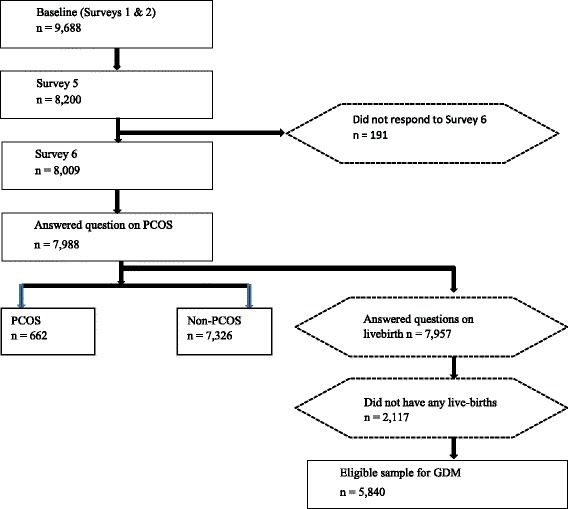
Consort diagram

This analysis focused on six waves of data from women in the 1973–1978 cohort, surveyed in 1996, 2000, 2003, 2006, 2009, and 2012. The eligible sample included all women who responded to Survey 6, as this reflects the most recent data (n = 8009), with the subgroup for GDM analysis including women with at least one live birth (n = 5840) (Fig. 1).

### Measures

#### BMI

BMI data were collected longitudinally in all surveys as height and weight based on self-reports. We computed group-based trajectory models across Surveys 2–6 to identify patterns in BMI change over time. We used five out of six BMI (83%) values on average.

#### PCOS

At Surveys 4, 5, and 6, women were asked “in the last three years, have you been diagnosed or treated for PCOS?”. Women who gave a positive response in any of the surveys where this question was asked were classified as having PCOS (coded as a binary variable). All women who responded negatively in any of surveys were considered as not having PCOS. Women who did not answer this question in any of the three surveys were excluded from the analysis.

#### GDM

Women were queried about the number of times that they had been diagnosed or treated for GDM and were considered to have a history of GDM (coded as a binary variable) if they reported at least one episode of GDM by Survey 6 (2012).

### Explanatory variables

#### Income

Women were asked to state their average household gross (before tax) income. Responses were categorized as < 15,999, 16,000–51,999, and ≥ 52,000 Australian dollars per annum.

#### Socioeconomic indexes for areas (SEIFA)

SEIFA was developed by the Australian Bureau of Statistics and ranks areas in Australia according to relative socioeconomic advantage and disadvantage. The indices were based on information from the 5-yearly censuses. The SEIFA index of education and occupation used here was designed to reflect the educational and occupational structure of communities at the collector’s district level. The indices are scored such that higher values indicate a greater advantage. As SEIFA was available from Survey 3 onwards, this was used in the analysis for GDM.

#### Educational qualification

Responses regarding the highest educational qualification completed were categorized as year 12 or lower, trade/certificate, and university/post-graduate degree.

#### Country of birth

Country of birth responses were categorized as a binary variable of Asian or other countries, including Australia, Africa, Europe, and other English speaking countries.

#### Smoking

Self-reported use of tobacco was categorized as non-smoker, ex-smoker, and current smoker.

#### Parity

The number of times a woman experienced a live birth was categorized as a binary variable of none or one or more live births.

### Statistical analysis

We used a χ^2^ test for categorical variables and independent Student’s *t* test for continuous data to describe the change in participant characteristics between PCOS and non-PCOS population subgroups. A *P* value less than 0.05 was considered a significant difference among the groups. GBTM across Surveys 2–6 was used to identify subpopulations of women following distinct patterns of BMI change over time, in the entire sample as well as in the PCOS and non-PCOS populations. Multinomial logistic regression was used to explore sociodemographic and health behavior characteristics associated with BMI trajectories. Variables statistically significantly associated with BMI trajectories in univariate analysis (*P* < 0.2), or considered potential confounders based on hypothesis testing or previous literature, were considered for inclusion in the final multivariate model. A binary logistic regression model was then used to assess the risk factors of GDM, especially regarding BMI trajectories and PCOS, adjusting for potential confounders such as SEIFA, occupation, and parity. We used a similar model-building strategy as outlined above. We obtained the final model to yield the most parsimonious subset of predictors that explained the data. We also tested for any interaction between PCOS and BMI trajectory group membership for the development of GDM. Although our model used complete case analysis, there was some missing data for the variable BMI collected over the six surveys. On exploratory analysis, the results did not suggest that the missing data conformed to a particular pattern, and we assumed the data to be missing at random. To understand the effect of missing data on BMI trajectory formation and its impact on the development of GDM among women with PCOS, we conducted sensitivity analyses using multiple imputations to understand how robust our results were. We conducted all analyses using Stata software version 14.0.

## Results

### Characteristics of study participants at Survey 6 according to PCOS status

Overall, 8009 women took part in the survey in 2012 (Survey 6), with a mean age of 36.7 years (SD 1.48) and a mean BMI of 26.4 kg/m^2^ (SD 6.30). More than half of the women had a university degree (53.3%) and 11.6% were current smokers. The vast majority of the population were of Australian, UK, and other English speaking backgrounds (97.9%), with only 1.46% of the population being of Asian descent. In total, 72.9% of these reproductive-aged women had a live birth by Survey 6.

By Survey 6, 7988 women answered the question on PCOS. There were no differences in educational qualification, SEIFA scores, ethnicity, and prevalence of smoking among women with PCOS compared to those without. However, women with PCOS displayed a significantly higher BMI (29.2 kg/m^2^ vs. 26.2 kg/m^2^) and a higher prevalence of GDM (14.1% vs. 7.42%, *P* < 0.001) at baseline (Table 1).

**Table 1:** Characteristics of study participants at Survey 6 (age 34–39 years) by PCOS status

Characteristics	Level	*N* (%)	*N* (%)	*N* (%)	*P* value^a^
		Whole group	PCOS	non-PCOS	
N		8009	662	7326	
Age, mean (SD)		36.7 (1.48)	36.6 (1.43)	36.8 (1.48)	0.001
BMI, mean (SD)		26.4 (6.30)	29.2 (7.86)	26.2 (6.07)	<0.001
Educational qualification	Year 12 or less	1421 (17.7)	106 (16.0)	1308 (17.9)	0.42
	Trade or certificate	2171 (27.1)	194 (29.3)	1973 (26.9)	
	University/post-graduate	4267 (53.3)	352 (53.2)	3910 (53.4)	
	Missing	150 (1.87)	10 (1.51)	135 (1.84)	
SEIFA mean (SD)		1015.1 (95.7)	1020.4 (98.3)	1014.6 (95.3)	0.14
Country of birth	Asian	117 (1.46)	14 (2.11)	103 (1.41)	0.15
	Australia/Other	7840 (97.9)	644 (97.3)	7175 (97.9)	
	Missing	52 (0.65)	4 (0.60)	48 (0.66)	
Smoking status	Non-smoker	4854 (60.6)	415 (62.7)	4432 (60.5)	0.36
	Past smoker	2167 (27.1)	180 (27.2)	1980 (27.0)	
	Current smoker	928 (11.6)	63 (9.52)	862 (11.8)	
	Missing	60 (0.75)	4 (0.60)	52 (0.71)	
Prevalence of GDM		460/5802 (7.93)	63/448 (14.1)	396/5339 (7.42)	<0.001
Prevalence of PCOS		662/7988 (8.29)			

^a^χ^2^ or *t* test used to compare PCOS and non-PCOS groups as appropriate

*BMI* body mass index, *GDM* gestational diabetes, *PCOS* polycystic ovary syndrome, *SD* standard deviation, *SEIFA* socioeconomic indexes for areas

### BMI trajectory groups

**Fig. 2:**
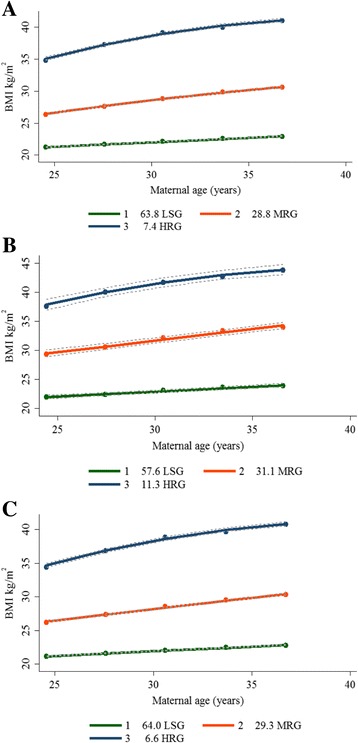
Latent-growth curve analyses used to identify distinct BMI trajectories by maternal age. **a** All women of reproductive age with BMI data. **b** Women with PCOS. **c** Women without PCOS. LSG (% of women in low-stable group); MRG (% of women in moderately rising group); HRG (% of women in high-rising group)

We determined BMI trajectories separately for all women of reproductive age (Fig. 2a) as well as for women with and without PCOS (Fig. 2b and c, respectively). When these trajectories were compared with existing World Health Organization (WHO) guidelines for classifying BMI, the following categories were identified. The low-stable group (LSG) included women who were characterized by an average trajectory that remained within the normal BMI range (<25 kg/m^2^) through ages 21–39 years. Their rate of growth showed a linear pattern of increase over time and was the largest group, at 63.8% of the population. The moderately rising group (MRG) was the second largest, at 28.8% of the population. This group started with their BMI in the overweight range (25 kg/m^2^) and remained within this category throughout the 13 years, ending at a BMI of approximately 30 kg/m^2^. Finally, the high-rising group (HRG) represented 7.40% of the population, starting and ending in the obese range, and had both a higher starting BMI and a steeper quadratic pattern of change in BMI over time. Among women with PCOS, there was a greater proportion who were characterized as belonging to both the overweight and obese (MRG and HRG) categories compared to those without PCOS and the entire sample of women of reproductive age (Fig. 2b, c). The models, however, did not identify a trajectory of decreasing BMI, nor a trajectory suggesting a change from MRG or HRG to LSG.

### Sociodemographic characteristics and relationship to BMI trajectories (whole population of reproductive-aged women)

We explored relationships between BMI trajectories and sociodemographic status among women of reproductive age (Table 2). Women characterized as MRG or HRG were more likely to have a higher starting BMI and be younger. Women of Asian descent had a higher tendency to be described as LSG compared to women of Australian or European ancestry. We were unable to conduct a similar assessment for HRG due to the small sample of Asian women available for this analysis. The sociodemographic characteristics did not differ among the three trajectory groups.

**Table 2:** Characteristics of BMI trajectory group membership

Characteristics		MRG			HRG		
		RRR	95% CI	*P* value	RRR	95% CI	*P* value^a^
BMI Survey 1 (kg/m^2^)		1.85	(1.78–1.91)	<0.001	2.92	(2.75–3.10)	<0.001
Age (years)		0.85	(0.80–0.90)	<0.001	0.66	(0.59–0.75)	<0.001
Smoking status	Ex-smoker	1.17	(0.94–1.46)	0.16	1.30	(0.83–2.05)	0.26
(ref non-smoker)	Current smoker	1.19	(0.99–1.42)	0.06	1.36	(0.93–1.98)	0.11
Exercise status	Low	1.27	(0.94–1.71)	0.13	0.78	(0.44–1.38)	0.39
(ref nil/sedentary)	Moderate	1.13	(0.83–1.54)	0.44	0.53	(0.29–0.99)	0.05
	High	0.84	(0.62–1.14)	0.27	0.46	(0.26–0.83)	0.01
Live birth		0.95	(0.80–1.13)	0.56	0.83	(0.58–1.18)	0.30
Country of birth	Asians	0.36	(0.16–0.79)	0.01	3.34 × 10^–7^	Not calculated	
(ref all other countries)							
Income (year 2)	$16,000–51,999	0.86	(0.69–1.06)	0.15	0.74	(0.49–1.12)	0.16
(ref < $15,999)	≥ $52,000	0.77	(0.58–1.02)	0.07	0.59	(0.32–1.10)	0.10
Education (ref < year 12)	Trade/certificate	1.13	(0.92–1.40)	0.25	0.83	(0.53–1.32)	0.43
	University/post-graduate	0.83	(0.65–1.06)	0.13	0.62	(0.34–1.11)	0.11

^a^Characteristics between PCOS and non-PCOS population were compared by χ^2^ or *t* test as appropriate

*BMI* body mass index, *CI* confidence interval, *HRG* high-rising group, *MRG* moderately rising group, *RRR* relative risk reduction

### BMI trajectories and PCOS status

There was a greater proportion of women with PCOS characterized as belonging to the overweight and obese (MRG and HRG) categories compared to those without and the entire sample of women of reproductive age (Fig. 2a–c). Women with PCOS were 1.55 times more likely to belong to the MRG trajectory and 4.66 times more likely to belong to the HRG trajectory. When we adjusted this association for well-known confounders, including parity, maternal age, and ethnicity (Asian), women with PCOS were 1.38 times more likely to belong to the MRG trajectory and 3.40 times more likely to belong to HRG trajectory, respectively (Table 3).

**Table 3:** PCOS status at Survey 6 associated with BMI trajectory group membership

Characteristics (Survey 6)		MRG			HRG		
		OR	95% CI	*P* value	OR	95% CI	*P* value
PCOS	(ref – no PCOS)	1.55	(1.29–1.86)	<0.001	4.66	(3.73–5.83)	<0.001
PCOS^a^	(ref – no PCOS)	1.38	(1.10–1.72)	0.01	3.40	(2.31–5.01)	<0.001

Multinomial regression used to compare the association between PCOS status at Survey 6 and BMI trajectory groups; Reference group: Low-stable group

^a^Adjusted for maternal age, baseline BMI, Asian ethnicity, and parity

*BMI* body mass index, *CI* confidence interval, *MRG* moderately rising group, *OR* odds ratio, *PCOS* polycystic ovary syndrome, *HRG* high-rising group

### BMI, PCOS, and GDM relationships

We undertook multivariate logistic regression to explore how BMI trajectory and PCOS were related to the prevalence of GDM. Both BMI trajectory and PCOS status were significantly associated with GDM. Compared to the LSG trajectory, women in the MRG (OR = 1.72, 95% CI 1.39–2.13) and HRG (OR = 2.50, 95% CI 1.80–3.48) trajectories were at an increased risk of developing GDM, independent of PCOS status, while those with PCOS were 1.89 times more likely to develop GDM independent of BMI change over time, parity, and SEIFA scores (Table 4). The obese BMI trajectory (HRG) was a stronger correlate of GDM than other traditional risk factors included in the model. We did not find any statistically significant interaction between BMI trajectory group membership and PCOS for the development of GDM (*P* = 0.27). On conducting sensitivity analysis by multiple imputations, associations between GDM and PCOS adjusting for BMI changed by approximately 22% and continued to remain significant (OR 2.08, 95% CI 1.98–2.66). We had, on average, five out of six BMI (83%) values available for trajectory formation, with the remaining values imputed during sensitivity analysis, suggesting that the initial results were robust for missing data over time.

**Table 4:** Independent characteristics associated with GDM

Characteristics	Crude OR 95% CI	*P* value	OR (adjusted)^a^ 95% CI	*P* value
PCOS	2.04 (1.54–2.72)	<0.001	1.89 (1.41–2.54)	<0.001
Trajectory groups				
LSG	1		1	<0.001
MRG	1.88 (1.53–2.31)	<0.001	1.72 (1.39–2.13)	
HRG	3.02 (2.21–4.12)	<0.001	2.50 (1.80–3.48)	
Maternal age, years	1.07 (1.01–1.15)	0.03	1.08 (1.01–1.16)	0.02
SEIFA	0.998 (0.997–0.999)	0.001	0.999 (0.998–1.00)	0.06
Parity (1–2 children)				
> 2 children	1.15 (0.93–1.41)	0.19	1.09 (0.88–1.35)	0.43

Logistic regression used to compare the association between GDM, PCOS, and BMI trajectory groups; Reference group: Women with no GDM

^a^Adjusted for maternal age, SEIFA, and parity

*CI* confidence interval, *GDM* gestational diabetes, *HRG* high-rising group, *LSG* low-stable group, *MRG* moderately rising group, *SEIFA* socioeconomic index for areas, *OR* odds ratio, *PCOS* polycystic ovary syndrome

## Discussion

Insulin-resistant conditions like obesity, PCOS, and GDM are increasing at an alarming rate in women of reproductive age in developed countries like Australia. Despite sharing IR as an underlying mechanism for the development of diabetes, the relationship between these conditions remains unclear. Here, we have generated novel longitudinal weight gain trajectories from a large community-based Australian cohort of women of reproductive age followed over 13 years. We show significantly distinct trajectories of weight gain predicted by early adult life (beginning of adult reproductive age) BMI. We also show a higher tendency to be characterized as LSG among women of Asian descent. We demonstrate that BMI trajectory is a stronger correlate of GDM than PCOS or other traditional confounders such as age, socioeconomic status, and parity.

Studies exploring developmental trajectories of BMI among women of reproductive age are scarce. In this novel longitudinal study, we discuss developmental trajectories of BMI among Australian women of reproductive age. We identified three rising BMI trajectories, namely LSG, MRG, and HRG. Our findings are relatively consistent with WHO cut-off points for classifying BMI, except for MRG, which began at a slightly lower BMI than the standard recommended value of 25 kg/m^2^, the existing WHO cut-off point for the overweight category [22]. Characterizing BMI trajectories in this cohort revealed that a higher early adult life BMI predicted membership in higher BMI trajectories. This highlights the differential impact of weight in predicting membership in high BMI trajectories. In contrast to prior research reporting significant correlations of socioeconomic factors with BMI, we did not find any significant association of income, education, or health behaviors, such as exercise and smoking, with BMI trajectory group membership [23, 24]. This could be because socioeconomic status and related factors, such as education and income, tend to remain relatively stable past a certain point in adulthood, possibly displaying less variability among this age cohort. Women of Asian descent were more likely to belong to the LSG compared to the MRG trajectory, consistent with prior literature reporting that women of Asian descent generally have a lower BMI [25] than Caucasian women, with central obesity rather than BMI being responsible for the metabolic impacts seen among South Asian women [26]. We were unable to explore similar associations among the HRG trajectory due to the small subgroup sample size and this should be studied further. Overall, this study highlights the need to target weight gain prevention in adolescents or young women of reproductive age.

Trajectories among women with and without PCOS, as well as within the entire sample of women of reproductive age, were similar, in terms of the number of trajectories as well as the observed growth patterns describing change in BMI over time. However, a greater proportion of women with PCOS were characterized as belonging to the overweight and obese (MRG and HRG) trajectories compared to the proportion of women without PCOS or those within the entire sample of women of reproductive age. Our findings are consistent with prior research reporting women with PCOS to have a higher baseline weight as well as an increased rate of weight gain over time compared to women without [18, 19].

BMI trajectories were independently associated with GDM development and were a stronger correlate than factors such as PCOS, maternal age, socioeconomic status, and parity. Prior evidence and meta-analyses also report an increased risk of GDM in overweight and obese women [6, 13, 27]. Our 2.50-fold increased risk among those in the HRG trajectory is greater than assessments of GDM adjusting for cross-sectional BMI, possibly reflecting the greater accuracy of using BMI trajectories in predicting the development of GDM. Given that our study findings suggest a predictive role of early adulthood BMI in predicting future BMI, interventions aimed at reducing adolescent and early reproductive life weight could be beneficial for IR-mediated diseases such as GDM. IR is one of the key pathophysiological features of obesity, PCOS, and GDM [8], independently of BMI. However, IR is further exacerbated by an increased BMI with obesity increasing the prevalence of GDM independent of PCOS [28]. Previous research has reported that the state of normal pregnancy induces a state of hyperinsulinemic IR [27], which, when compounded with baseline IR observed among obese women, as well as women with PCOS, may amplify the absolute risk of pregnancy complications [29] and contribute to a higher risk of GDM among this subgroup.

The main strengths of the study include the large, unselected community cohort with a good retention of participants over time, limited information bias, and the ability to adjust for a number of important confounders. ALSWH staff regularly compare the most recent census data and data from national health surveys with corresponding data from ALSWH surveys to enable them to document the extent to which representativeness is maintained and to quantify biases that might affect the generalizability of findings [30]. A recent comparison of women who participated in the baseline ALSWH survey with women from the Australian 1996 census within the same age range showed participants to be representative of the general population, underscoring the generalizability of our findings for Australia and similar contexts [31].

Limitations include the use of self-reported measures of BMI, PCOS, and GDM. However, self-reported measures of PCOS have been validated with menstrual irregularity among this cohort of women. Self-reported BMI, as used in this study, has been validated with anthropometric measurement among the mid-age cohort from ALSWH. Further, the self-reported measure of GDM used in this study has also been validated by Gresham et al. [32] against objective medical records from the New South Wales Perinatal Data Collection, showing very high validity (≥92%) and reliability between the two datasets. Another limitation is that we do not know the specific timing of GDM and T2DM within the surveys. In Survey 6, it is possible for women to have had GDM and then post-partum T2DM within the 3-year period since the last survey. Thus, we have not excluded these women from our analysis, but acknowledge that we do not know the specific timing of these two events. Finally, it is unclear if the young cohort is representative of Asian women given the very low numbers of Asian women participating in the study. This was most likely due to lower immigration rates of women from non-English speaking countries before 1996, as the cohort did not include women who arrived in Australia after 1996 [33].

## Conclusion

Our results indicate heterogeneity in BMI development among young Australian women over the reproductive years and advance our understanding of longitudinal change in BMI both among women with and without PCOS. Our findings highlight that the risk of a high BMI trajectory can be detected at an young age, both in women with or without PCOS. This presents opportunities for intervention and emphasizes the need to focus on early prevention of weight gain. Women with PCOS were more likely to be in the high-rising BMI group; therefore, prevention here is even more important. BMI trajectory was a stronger correlate of GDM than PCOS and other conventional risk factors, further emphasizing the importance of prevention of weight gain. Women with a history of PCOS would also benefit from early screening for GDM and lifestyle intervention from early pregnancy to avoid excess gestational weight gain and maintain a healthy lifestyle throughout pregnancy. Early adult weight, modifiable through lifestyle change, was found to predict subsequent BMI trajectories for Australian women of reproductive age. Our findings suggest an ideal opportunity to intervene at an early stage of reproductive life [34] and reduce the risk of long-term weight-related health sequelae such as GDM. Our findings need to be replicated in different populations for confirmation, particularly among Asian women, to generalize these findings.
